# Invasive and Non-invasive Assessment of Non-culprit Coronary Lesions in Patients with ST-segment Elevation Myocardial Infarction

**DOI:** 10.37825/2239-9747.1050

**Published:** 2024-05-02

**Authors:** Germano Ferruzzi, Michele Bellino, Angelo Silverio, Marco Di Maio, Mariagiovanna Vassallo, Carmine Vecchione, Gennaro Galasso

**Affiliations:** a Department of Medicine, Surgery and Dentistry, University of Salerno, Baronissi, Salerno, Italy; b Vascular Physiopathology Unit, IRCCS Neuromed, Pozzilli, Italy

**Keywords:** Acute coronary syndrome, Non-culprit lesion, Multivessel disease, Intracoronary imaging, IVUS, OCT, Functional assessment, Coronary computed tomography angiography

## Abstract

The angiographic evidence of coronary multivessel disease (MVD) increases significantly the risk of recurrent ischemic events in patients with ST-segment elevation myocardial infarction (STEMI).

Recent evidence suggests that a complete revascularization strategy should be considered the standard of care in these patients and performed for significant non-culprit lesions (NCLs) after careful assessment of the individual risk-benefit ratio. However, the optimal timing and the modality for the assessment of NCLs is not fully standardized.

This brief review aims to summarise the management of MVD in patients with STEMI and to provide an overview of the principal techniques used to guide revascularisation in this high-risk clinical setting.

## 1. Introduction

A bout half of patients with ST-segment elevation myocardial infarction (STEMI) exhibits coronary multivessel disease (MVD), defined as the presence of two or more epicardial coronary arteries with obstructive luminal narrowing [1]. The angiographic evidence of MVD has achieved increasing clinical interest in the last decades due to the robust evidence of its association with recurrent ischemic events and mortality [2–5].

Current guidelines recommend routine revascularization of non-culprit lesions(NCLs),definedas any coronary lesion not responsible for the acute coronary syndrome (ACS), during the index percutaneous coronary intervention (PCI) procedure or within 45 days in haemodynamically stable patients [6].

The decision to perform coronary revascularisation needs to be carefully evaluated against the risks of the procedure and the patient’s comorbidities, especially in the case of complex coronary lesions [7–11].

Therefore, the identification of really significant NCLs is central to the decision-making process. No one technique fits all patients, but the integration of invasive and non-invasive techniques is currently considered the most rational approach by the interventional cardiology community [12,13].

This short review aims to summarise the management of MVD in patients with STEMI and to provide an overview of the techniques used to guide revascularisation decisions.

## 2. Treatment of non-culprit lesions

Robust evidence of NCLs treatment in patients with ACS and MVD demonstrated the safety and effectiveness of complete revascularization and its value in reducing the risk of long-term adverse events (Table 1) [14–18]. The Preventive Angioplasty in Myocardial Infarction (PRAMI) trial included 465 patients with STEMI and MVD, who were randomized to immediate angiography-guided multivessel complete revascularization versus culprit-only lesion primary PCI [7]. The study demonstrated the benefit of immediate multivessel PCI with a significant reduction of the composite endpoint including cardiovascular death, non-fatal myocardial infarction or refractory angina at three years follow-up, as compared to culprit-only lesion PCI [14].

Consistently with this study, the Complete versus Lesion-only Primary PCI (CvLPRIT) trial showed a significantly lower risk for the composite of all-cause mortality, recurrent myocardial infarction, heart failure, and ischemia-driven revascularization at one-year follow-up in patients with STEMI and MVD undergoing early angiography-guided complete revascularization compared to culprit-only lesion PCI [15]. Also, the Treatment of Culprit Lesion Only or Complete Revascularization (DANAMI-3 PRIMULTI), the FFR Guided Revascularization Versus Conventional Strategy in Acute STEMI Patients With MVD (COMPARE-ACUTE) and the Complete vs. Culprit-only Revascularization to Treat Multi-vessel Disease After Early PCI for STEMI (COMPLETE) trials demonstrated the benefit of complete revascularization guided by early fractional flow reserve (FFR) or coronary angiography compared to culprit-only lesion primary PCI [16–18].

The mechanisms underlying the association between NCLs and adverse cardiac events are complex and multifactorial. In the acute phase of STEMI, the release of inflammatory substances promotes instability of NCLs and microcirculatory endothelial damage potentially leading to thrombosis, plaque rupture and myocardial ischemia [19–23]. After the acute phase, NCLs may undergo disease progression, especially in patients with multiple and not optimally controlled cardiovascular risk factors such as diabetes, high cholesterol levels, hypertension, smoking, and chronic kidney disease [24–29].

The optimal timing to complete myocardial revascularization is controversial, since some trials treated NCLs during index PCI [14,15] and others planned revascularization in staged procedure [16,18].

The Direct Complete Versus Staged Complete Revascularization in Patients Presenting With Acute Coronary Syndromes and Multivessel Disease (BIO-VASC) trial enrolled 764 patientswith ACS, who were randomized to immediate vs staged complete multivessel PCI. The study showed that complete revascularisation at index PCI was not inferior to staged PCI in terms of the composite of all-cause mortality, myocardial infarction, any unplanned ischemia-driven revascularization, or cerebrovascular events. Moreover, immediate complete PCI of NCLs was associated with a reduction in myocardial infarction and unplanned ischaemic-driven revascularisation [30].

Consistently with this study, MULTivessel Immediate versus STAged RevaScularization in Acute Myocardial Infarction (MULTISTARS AMI) trial compared the safety and efficacy of immediate vs staged complete revascularisation in haemodynamically stable patients with STEMI and MVD. The results showed that achieving complete revascularisation during index PCI was not inferior to staged PCI in terms of mortality and ischaemic events [31].

## 3. Invasive assessment

### 3.1. Coronary physiology

Although previous studies have demonstrated the potential benefit of invasive functional assessment for PCI guidance in patients with stable ischemic heart disease [32,33], this evidence cannot be extended to patients with ACS.

FFR is the most used technique to evaluate the functional severity of coronary lesions during adenosine-induced hyperemia. Non-hyperaemic pressure ratios (NHPRs) provide an alternative method for evaluating the ratio between distal coronary pressure and aortic pressure across coronary lesions during the wave-free period of the resting cardiac cycle without the need for hyperaemic stimuli [34].

In the acute or subacute phase of ACS, the release of inflammatory mediators, distal embolization, vasoconstriction of microcirculation and increased left ventricular tele-diastolic pressure may promote a transient change in coronary physiology and thus affect measurements of both hyperaemic- and non-hyperaemic-based indexes [35–39].

Two randomized clinical trials investigated the role of complete revascularization guided by invasive functional vs angiography strategy in patients with ACS and MVD (Table 2) [38,40].

The FLOW Evaluation to Guide Revascularization in Multi-vessel ST-elevation Myocardial Infarction (FLOWER-MI) trial enrolled 1171 patients with STEMI and MVD, who were randomized to receive complete revascularization guided by FFR or angiography alone. At one-year, complete revascularization guided by FFR did not show significant benefit compared to revascularization guided by angiography alone [38]. However, the unexpected very low rate of adverse events during follow-up makes the results of this study not conclusive [38]. More recently, the Fractional flow reserve versus angiography-guided strategy in acute myocardial infarction with multivessel disease (FRAME-AMI) trial demonstrated a lower risk of the composite of death, myocardial infarction, or repeat revascularization in patients with ACS and MVD undergoing complete revascularization guided by FFR compared to PCI guided by angiography strategy [40].

Therefore, the implementation of physiology to guide PCI decisions of NCLs remains controversial.

### 3.2. Intravascular imaging

The assessment of NCLs using intravascular imaging techniques can help to identify plaque characteristics associated with a higher risk of rupture including a high percentage of lipids, necrotic core, and thin fibrous cap (tin-cap fibroatheroma, TCFA), in patients with STEMI and MVD (Table 2) [41–43].

Currently, the main catheter-based imaging techniques are: intravascular ultrasound (IVUS), near-infrared spectroscopy (NIRS) and optical coherence tomography (OCT).

IVUS is an intravascular coronary imaging technique based on the use of ultrasounds with high penetration depth, which allows the assessment of the plaque burden and of the minimal lumen area (MLA) [42]. The Providing Regional Observations to Study Predictors of Events in the Coronary Tree (PROSPECT) study enrolled 697 patients with ACS, who underwent three-vessel IVUS for the assessment of NCLs. The authors showed that the presence of plaque burden ≥70%, the presence of TCFA and an MLA ≤4 mm [2] were independent predictors of adverse events [19].

The assessment of lipid-rich plaques may be facilitated through NIRS, which is an intravascular coronary imaging technique used to estimate the lipid pool extension in coronary lesions [43,44]. In Near-infrared spectroscopy predicts cardiovascular outcome in patients with coronary artery disease (ATHEROREMO-NIRS) study, lipid-rich plaques were associated with cardiovascular adverse events during follow-up [44]. More recently, NIRS has been implemented in IVUS catheters to evaluate simultaneously the plaque burden and its lipid percentage [43]. Indeed, the high percentage of lipid in coronary lesion and the high plaque burden has been associated with a significantly higher risk of adverse cardiac events [43].

Optical coherence tomography (OCT) is characterized by higher spatial resolution and allows a more accurate estimation of the thickness of TCFA and the detection of plaque components [45]. In the Relationship between coronary plaque morphology of the left anterior descending artery and 12 months clinical outcome (CLIMA) study, the authors investigated the predictive value of specific plaque features identified by OCT. Among 1776 coronary lesions, the presence of MLA<3.5 mm2, fibrous cap thickness <75 μm, lipid arc circumferential extension> 180, and macrophage infiltration were associated with a higher risk of cardiovascular events [46]. Also, in the Thin-cap fibroatheroma predicts clinical events in diabetic patients with normal fractional flow reserve (COMBINE OCT-FFR) trial, the presence of TCFA was associated with poor outcome in diabetic patients with negative FFR [47].

Therefore, intravascular imaging may help to identify characteristics of plaque vulnerability in NCLs and may improve the prognostic stratification in patients with STEMI and MVD. However, the optimal revascularization strategy for patients with vulnerable NCLs remains to be determined.

## 4. Non-invasive assessment

The timing and the modality for non-invasive evaluation of residual ischemia in patients with NCLs and recent STEMI is still a matter of debate and depends largely on the local availability and expertise [6].

In addition, coronary computed tomography angiography (CCTA) may be implemented to identify specific features of atherosclerotic plaques such as low-attenuation plaque, napkin-ring sign, positive remodeling and spotty plaque calcification, which are associated with a higher risk of further adverse events (Table 2) [48–51]. Ferencik and colleagues, in a secondary analysis of the PROMISE trial including 4415 patients, showed that high-risk plaques as defined by CCTA were associated with cardiovascular adverse events during follow-up. However, the study showed a low positive predictive value for CCTA which limits its clinical perspectives [52].

Currently, CCTA has been combined with computational fluid dynamics technologies to evaluate simultaneously both anatomical features and functional information of atherosclerotic plaques [53,54].

Lee et al. evaluated the prognostic significance of non-invasive hemodynamic parameters using CCTA in patients with NCLs and recent ACS. The study suggested that integrating non-invasive haemodynamic information with anatomical plaque characterization may improve the identification of NCLs with the highest risk of adverse events [55]. However, the diagnostic accuracy of non-invasive hemodynamic parameters by CCTA for detecting residual ischemia in patients with NCLs and recent ACS was only modest when compared to invasive physiology techniques [56].

Cardiac magnetic resonance (CMR) is another non-invasive imaging modality that allows simultaneous assessment of left ventricular morphology and function, tissue characterization, and stress myocardial perfusion [57,58].

A recent sub-study from the Reducing Micro Vascular Dysfunction in Acute Myocardial Infarction by Ticagrelor (REDUCE-MVI) trial including 77 patients demonstrated the usefulness of stress perfusion CMR with adenosine for detecting residual ischemia in patients with NCLs and recent ACS. However, the diagnostic performance of this noninvasive technique was moderate when compared to invasive FFR assessment at one month (Table 2) [59].

Currently, the main limitation to the implementation of non-invasive diagnostic tools for the assessment of NCLs in patients with recent STEMI is the modest positive predictive value for the identification of lesions associated with a higher risk of future adverse events compared to invasive techniques [56,59]. However, these methods are virtually risk-free and have the potential to avoid invasive examinations in patients with non-significant NCLs who do not require further coronary revascularization.

Future studies will determine whether non-invasive methods can be employed to guide the decision-making in STEMI patients with MVD.

## 5. Future perspectives

Currently, the optimal strategy for defining the anatomical and hemodynamic significance of NCLs to guide complete revascularisation decisions in patients with STEMI and coexisting NCLs remains to be determined.

Currently, the application of invasive physiology measurements to guide decisions on PCI of NCLs remains controversial. Therefore, the Physiology-guided vs Angiography-guided Non-culprit Lesion Complete Revascularization for Acute MI & Multivessel Disease (COMPLETE-2) study, a phase 3 trial, will investigate the prognostic role of physiology-guided complete revascularization compared to conventional angiography strategy in patients with MVD and recent ACS.

The Preventive PCI or medical therapy alone for vulnerable atherosclerotic coronary plaque (PREVENT) study will investigate the prognostic impact of preventive PCI in patients with high-risk plaque features quantified by intravascular imaging and negative value of FFR (FFR >0.80), compared to medical therapy alone.

## 6. Conclusions

In patients with STEMI, the prevalence of MVD is high and modern evidence supports the complete revascularization strategy during the index PCI procedure or at least within the first month to improve the patients’ short- and long-term outcomes [6].

What is the best invasive or non-invasive modality for assessing the clinical relevance of NCLs remains an open question. In daily practice, the assessment of patients’ comorbidities, hemodynamic status and coronary anatomy, together with the experience of the individual center in percutaneous treatment of complex lesions, directs patients toward dedicated pathways of clinical management.

Further evidence from large randomized trials is needed to establish a standardized approach in this complex clinical scenario.

## Figures and Tables

**Table 1:** Randomized clinical trials comparing complete revascularization vs. culprit-only lesion revascularization strategies.

Study, years	Sample size	NCLs severity	Primary outcome	Main result
PRAMI, 2013	465	angiographic diameter stenosis >50%	MACE: CV death, non-fatal MI, refractory angina at 23 months FU	Complete multivessel PCI of NCLs was associated with reduced risk of MACE compared to culprit-only PCI
CvLPRIT, 2015	296	Angiography diameter stenosis >70% or >50% in 2 views	MACE: Death, MI, any repeat revascularization, HF at 12 months FU.	Complete multivessel PCI was associated with lower risk of MACE compared to culprit-only PCI
DANAMI-3 PRIMULTI, 2015	627	Angiography diameter stenosis >90% or Angiography diameter stenosis >50% and FFR<0.80	MACE: Death, re-infarction, ischemia-driven revascularization at 27 months FU.	Complete multivessel PCI guided by FFR reduced the risk of MACE compared with no further invasive intervention after primary PCI
COMPARE-ACUTE, 2017	885	Angiography diameter stenosis>50% and FFR<0.80	MACE: Death, non-fatal MI, revascularization, cerebrovascular events at 12 months FU	Complete multivessel PCI guided by FFR reduced the risk of MACE compared to culprit-only PCI
COMPLETE, 2019	4041	Angiography diameter stenosis >70% or angiography diameter stenosis between 50% and 69% and FFR <0.80	Coprimary outcome 1. Composite of CV death and MI 2. Composite of CV death, MI, and ischemia-driven revascularization at 36 months FU	Complete multivessel PCI reduced the risk of both coprimary outcome compared to culprit-only PCI

CV, cardiovascular; FFR, fractional flow reserve; FU, follow-up; HF, heart failure; MI, myocardial infarction; MACE, major adverse cardiac events; MVD, multivessel disease; NCLs, non-culprit lesions; PCI, percutaneous coronary intervention.

**Table 2:** Studies providing the invasive and non-invasive assessment of NCLs in patients with multivessel coronary disease.

Study, years	Sample size	NCLs severity	Primary outcome	Main result
**Coronary physiology**				
FLOWER-MI, 2021	1171	Angiographic diameter stenosis >50% vs Angiography diameter stenosis >50% and FFR<0.80	MACE: Death, non-fatal MI, unplanned hospitalization leading to urgent revascularization at 12 months FU	Complete multivessel PCI guided by FFR did not show significant benefit compared to PCI guided by angiography alone
FRAME-AMI, 2022	562	Angiographic diameter stenosis >50% vs Angiography diameter stenosis >50% and FFR<0.80	MACE: Death, MI and repeat revascularization, at 41 months FU.	Complete multivessel PCI guided by FFR reduced the risk of MACE compared to PCI guided by angiography alone
**Intravascular imaging**				
PROSPECT, 2011	697	Three-vessel IVUS for the assessment of plaque features	MACE: CV death, cardiac arrest, MI, rehospitalization due to unstable or progressive angina at 36 months FU.	The presence of plaque burden ≥70%, TCFA and an MLA ≤4mm2 were independent predictors of MACE
ATHEROREMO-NIRS, 2014	203	NIRS for the assessment of lipid-rich plaques	MACE: Death, non-fatal MI, stroke, unplanned coronary revascularization at 12 months FU	The presence of lipid-rich plaques was associated with higher risk of MACE
CLIMA, 2020	1003	OCT for the assessment of plaque features	MACE: cardiac death and target segment myocardial infarction at 12 months FU	MLA<3.5 mm^2^, fibrous cap thickness <75 μm, lipid arc circumferential extension>180, and macrophage infiltration were associated with a higher risk of MACE
COMBINE OCT-FFR, 2021	550	OCT detected TCFA in diabetes patients with negative FFR value	MACE: CV death, target vessel MI, clinically driven target lesion revascularization or hospitalization due to unstable or progressive angina at 18 months	The presence of TCFA was associated with higher risk of MACE
**Non-invasive diagnostic tools**				
PROMISE, 2018	4415	CCTA for the assessment of high-risk plaque features	MACE: death, myocardial infarction, or unstable angina at 25 months	Positive remodelling, low computed tomographic attenuation, or napkin-ring sign were associated with a higher risk of MACE
REDUCE-MVI sub-study, 2020	77	Stress perfusion CMR with adenosine Vs FFR<0.80	The agreement between CMR and invasive FFR in the assessment of NCLs at 1 month	The diagnostic performance of CMR was moderate when compared to invasive FFR assessment

CCTA, coronary computed tomography angiography; CMR, cardiac magnetic resonance; CV, cardiovascular; FFR, fractional flow reserve; FU, follow-up; IVUS, intravascular ultrasound; MI, myocardial infarction; MACE, major adverse cardiac events; MLA, minimal lumen area; MVD, multivessel disease; NCLs, non-culprit lesions; OCT, Optical coherence tomography; PCI, percutaneous coronary intervention; TFCA, tin-cap fibroatheroma.

## Abbreviations

ACS: acute coronary syndrome
CCTA: coronary computed tomography angiography
CMR: cardiac magnetic resonance
FFR: fractional flow reserve
IVUS: intravascular ultrasound
MLA: minimal lumen area
MVD: multivessel coronary disease
NCLs: non-culprit lesions
NHPRs: non-hyperaemic pressure ratios
NIRS: near-infrared spectroscopy
NSTEMI: non-ST-segment elevation myocardial infarction
OCT: optical coherence tomography
PCI: percutaneous coronary intervention
STEMI: ST-segment elevation myocardial infarction
TFCA: tin-cap fibroatheroma

## References

[1] Park DW Clare RM Schulte PJ Pieper KS Shaw LK Califf RM Extent, location, and clinical significance of non-infarct-related coronary artery disease among patients with ST-elevation myocardial infarction JAMA 2014 312 2019 22 25399277 10.1001/jama.2014.15095

[2] Sorajja P Gersh BJ Cox DA McLaughlin MG Zimetbaum P Impact of multivessel disease on reperfusion success and clinical outcomes in patients undergoing primary percutaneous coronary intervention for acute myocardial infarction Eur Heart J 2007 28 1709 16 17556348 10.1093/eurheartj/ehm184

[3] Parodi G Memisha G Valenti R Trapani M Migliorini A Santoro GM Five year outcome after primary coronary intervention for acute ST elevation myocardial infarction: results from a single center experience Heart 2005 91 1541 4 15814595 10.1136/hrt.2004.054692 PMC1769226

[4] Galasso G De Angelis E Silverio A Di Maio M Cancro FP Esposito L Predictors of recurrent ischemic events in patients with ST-segment elevation myocardial infarction Am J Cardiol 2021 159 44 51 34503819 10.1016/j.amjcard.2021.08.019

[5] Di Maio M Esposito L Silverio A Bellino M Cancro FP De Luca G Prognostic significance of the SYNTAX score and SYNTAX score II in patients with myocardial infarction treated with percutaneous coronary intervention Catheter Cardiovasc Interv 2023 Nov 102 5 779 87 37702117 10.1002/ccd.30842

[6] Byrne RA Rossello X Coughlan JJ Barbato E Berry C Chieffo A ESC Scientific Document Group 2023 ESC Guidelines for the management of acute coronary syndromes Eur Heart J 2023 Aug 25 ehad191

[7] Silverio A Cancro FP Esposito L Bellino M D’Elia D Verdoia M Secondary cardiovascular prevention after acute coronary syndrome: emerging risk factors and novel therapeutic targets J Clin Med 2023 Mar 10 12 6 2161 36983163 10.3390/jcm12062161 PMC10056379

[8] Silverio A Di Maio M Buccheri S De Luca G Esposito L Sarno G Validation of the academic research consortium high bleeding risk criteria in patients undergoing percutaneous coronary intervention: a systematic review and meta-analysis of 10 studies and 67,862 patients Int J Cardiol 2022 Jan 15 347 8 15 34774882 10.1016/j.ijcard.2021.11.015

[9] Baldi C Silverio A Esposito L Di Maio M Tarantino F De Angelis E Clinical outcome of patients with ST-elevation myocardial infarction and angiographic evidence of coronary artery ectasia Catheter Cardiovasc Interv 2022 Feb 99 2 340 7 33949766 10.1002/ccd.29738 PMC9541104

[10] Silverio A Buccheri S Venetsanos D Alfredsson J Lagerqvist B Persson J Percutaneous treatment and outcomes of small coronary vessels: a SCAAR report JACC Cardiovasc Interv 2020 Apr 13 13 7 793 804 32061601 10.1016/j.jcin.2019.10.062

[11] De Luca G Silverio A Verdoia M Siudak Z Tokarek T Kite TA Angiographic and clinical outcome of SARS-CoV-2 positive patients with ST-segment elevation myocardial infarction undergoing primary angioplasty: a collaborative, individual patient data meta-analysis of six registry-based studies Eur J Intern Med 2022 Nov 105 69 76 35999094 10.1016/j.ejim.2022.08.021 PMC9385833

[12] Bellino M Silverio A Esposito L Cancro FP Ferruzzi GJ Di Maio M Moving toward precision medicine in acute coronary syndromes: a multimodal assessment of nonculprit lesions J Clin Med 2023 Jul 7 12 13 4550 37445584 10.3390/jcm12134550 PMC10343016

[13] Silverio A Zilio F Ciliberti G Paolisso P Barbato E Definizione, classificazione e diagnosi di infarto miocardico in assenza di coronaropatia ostruttiva: a che punto siamo? [Definition, classification and diagnosis of myocardial infarction with non-obstructive coronary artery disease: where do we stand?] G Ital Cardiol 2023 Oct 24 10 29 36 (Italian) 10.1714/4101.40992 37767845

[14] Wald DS Morris JK Wald NJ Chase AJ Edwards RJ Hughes LO Randomized trial of preventive angioplasty in myocardial infarction N Engl J Med 2013 369 1115 23 23991625 10.1056/NEJMoa1305520

[15] Gershlick AH Khan JN Kelly DJ Greenwood JP Sasikaran T Curzen N Randomized trial of complete versus lesion-only revascularization in patients undergoing primary percutaneous coronary intervention for STEMI and multivessel disease: the CvLPRIT trial J Am Coll Cardiol 2015 65 963 72 25766941 10.1016/j.jacc.2014.12.038 PMC4359051

[16] Engstrøm T Kelbæk H Helqvist S Høfsten DE Kløvgaard L Holmvang L Complete revascularisation versus treatment of the culprit lesion only in patients with ST-segment elevation myocardial infarction and multivessel disease (DANAMI-3–PRIMULTI): an open-label, randomised controlled trial Lancet 2015 386 665 71 26347918 10.1016/s0140-6736(15)60648-1

[17] Smits PC Abdel-Wahab M Neumann FJ Boxma-de Klerk BM Lunde K Schotborgh CE Fractional flow reserve-guided multivessel angioplasty in myocardial infarction N Engl J Med 2017 Mar 30 376 13 1234 44 28317428 10.1056/NEJMoa1701067

[18] Mehta SR Wood DA Storey RF Mehran R Bainey KR Nguyen H Complete revascularization with multivessel PCI for myocardial infarction N Engl J Med 2019 Oct 10 381 15 1411 21 31475795 10.1056/NEJMoa1907775

[19] Stone GW Maehara A Lansky AJ de Bruyne B Cristea E Mintz GS A prospective natural-history study of coronary atherosclerosis N Engl J Med 2011 Jan 20 364 3 226 35 21247313 10.1056/NEJMoa1002358

[20] Heusch G Libby P Gersh B Yellon D Böhm M Lopaschuk G Cardiovascular remodelling in coronary artery disease and heart failure Lancet 2014 May 31 383 9932 1933 43 24831770 10.1016/S0140-6736(14)60107-0 PMC4330973

[21] Konijnenberg LSF Damman P Duncker DJ Kloner RA Nijveldt R van Geuns RM Pathophysiology and diagnosis of coronary microvascular dysfunction in ST-elevation myocardial infarction Cardiovasc Res 2020 Mar 1 116 4 787 805 31710673 10.1093/cvr/cvz301 PMC7061278

[22] Esposito L Cancro FP Silverio A Di Maio M Iannece P Damato A COVID-19 and acute coronary syndromes: from pathophysiology to clinical perspectives Oxid Med Cell Longev 2021 Aug 30 2021 4936571 10.1155/2021/4936571 PMC8410438 34484561

[23] De Rosa R Silverio A Varricchio A De Luca G Di Maio M Radano I Meta-analysis comparing outcomes after everolimus-eluting bioresorbable vascular scaffolds versus everolimus-eluting metallic stents in patients with acute coronary syndromes Am J Cardiol 2018 Jul 1 122 1 61 8 29699747 10.1016/j.amjcard.2018.03.003

[24] Libby P Mechanisms of acute coronary syndromes and their implications for therapy N Engl J Med 2013 May 23 368 21 2004 13 23697515 10.1056/NEJMra1216063

[25] Bangalore S Toklu B Feit F Outcomes with coronary artery bypass graft surgery versus percutaneous coronary intervention for patients with diabetes mellitus: can newer generation drug-eluting stents bridge the gap? Circ Cardiovasc Interv 2014 Aug 7 4 518 25 24939927 10.1161/CIRCINTERVENTIONS.114.001346

[26] Silverio A Cancro FP Di Maio M Bellino M Esposito L Centore M Lipoprotein(a) levels and risk of adverse events after myocardial infarction in patients with and without diabetes J Thromb Thrombolysis 2022 Oct 54 3 382 92 36125640 10.1007/s11239-022-02701-w PMC9553824

[27] Scudiero F Canonico ME Sanna GD Dossi F Silverio A Galasso G Dual antiplatelet therapy with 3rd generation P2Y12 inhibitors in STEMI patients: impact of body mass index on loading dose-response Cardiovasc Drugs Ther 2023 Aug 37 4 695 703 35175499 10.1007/s10557-022-07322-2

[28] Bellino M Galasso G Silverio A Tedeschi M Formisano C Romei S Soluble PCSK9 inhibition: indications, clinical impact, new molecular insights and practical approach-where do we stand? J Clin Med 2023 Apr 18 12 8 2922 37109259 10.3390/jcm12082922 PMC10146045

[29] Silverio A Di Maio M Prota C De Angelis E Radano I Citro R Safety and efficacy of non-vitamin K antagonist oral anticoagulants in elderly patients with atrial fibrillation: systematic review and meta-analysis of 22 studies and 440 281 patients Eur Heart J Cardiovasc Pharmacother 2021 Apr 9 7 FI1 f20 9 31830264 10.1093/ehjcvp/pvz073

[30] Diletti R den Dekker WK Bennett J Schotborgh CE van der Schaaf R Sabaté M Immediate versus staged complete revascularisation in patients presenting with acute coronary syndrome and multivessel coronary disease (BIOVASC): a prospective, open-label, non-inferiority, randomised trial Lancet 2023 Apr 8 401 10383 1172 82 36889333 10.1016/S0140-6736(23)00351-3

[31] Stähli BE Varbella F Linke A Schwarz B Felix SB Seiffert M Timing of complete revascularization with multivessel PCI for myocardial infarction N Engl J Med 2023 Oct 12 389 15 1368 79 37634190 10.1056/NEJMoa2307823

[32] De Bruyne B Pijls NH Kalesan B Barbato E Tonino PA Piroth Z Fractional flow reserve-guided PCI versus medical therapy in stable coronary disease N Engl J Med 2012 Sep 13 367 11 991 1001 10.1056/NEJMoa1205361. Epub 2012 Aug 27. Erratum in: N Engl J Med 2012 Nov;367(18):1768 22924638

[33] Parikh RV Liu G Plomondon ME Sehested TSG Hlatky MA Waldo SW Utilization and outcomes of measuring fractional flow reserve in patients with stable ischemic heart disease J Am Coll Cardiol 2020 Feb 4 75 4 409 19 32000953 10.1016/j.jacc.2019.10.060

[34] Neumann FJ Sousa-Uva M Ahlsson A Alfonso F Banning AP Benedetto U ESC Scientific Document Group 2018 ESC/EACTS Guidelines on myocardial revascularization Eur Heart J 2019 Jan 7 40 2 87 165 Erratum in: Eur Heart J 2019 Oct 1;40(37):3096 30165437

[35] Teunissen PF Timmer SA Danad I de Waard GA van de Ven PM Raijmakers PG Coronary vasomotor function in infarcted and remote myocardium after primary percutaneous coronary intervention Heart 2015 Oct 101 19 1577 83 26246402 10.1136/heartjnl-2015-307825

[36] Thim T van der Hoeven NW Musto C Nijveldt R Götberg M Engstrøm T Evaluation and management of nonculprit lesions in STEMI JACC Cardiovasc Interv 2020 May 25 13 10 1145 54 32438985 10.1016/j.jcin.2020.02.030

[37] Thim T Götberg M Fröbert O Nijveldt R van Royen N Baptista SB Nonculprit stenosis evaluation using instantaneous wave-free ratio in patients with ST-segment elevation myocardial infarction JACC Cardiovasc Interv 2017 Dec 26 10 24 2528 35 29198461 10.1016/j.jcin.2017.07.021

[38] Puymirat E Cayla G Simon T Steg PG Montalescot G Durand-Zaleski I Multivessel PCI guided by FFR or angiography for myocardial infarction N Engl J Med 2021 Jul 22 385 4 297 308 33999545 10.1056/NEJMoa2104650

[39] Esposito L Di Maio M Silverio A Cancro FP Bellino M Attisano T Treatment and outcome of patients with coronary artery ectasia: current evidence and novel opportunities for an old dilemma Front Cardiovasc Med 2022 Feb 4 8 805727 35187112 10.3389/fcvm.2021.805727 PMC8854288

[40] Lee JM Kim HK Park KH Choo EH Kim CJ Lee SH Fractional flow reserve versus angiography-guided strategy in acute myocardial infarction with multivessel disease: a randomized trial Eur Heart J 2023 Feb 7 44 6 473 84 36540034 10.1093/eurheartj/ehac763

[41] Virmani R Burke AP Farb A Kolodgie FD Pathology of the vulnerable plaque J Am Coll Cardiol 2006 Apr 18 47 8 Suppl C13 8 16631505 10.1016/j.jacc.2005.10.065

[42] Xie Y Mintz GS Yang J Doi H Iñiguez A Dangas GD Clinical outcome of nonculprit plaque ruptures in patients with acute coronary syndrome in the PROSPECT study JACC Cardiovasc Imaging 2014 Apr 7 4 397 405 24631511 10.1016/j.jcmg.2013.10.010

[43] Erlinge D Maehara A Ben-Yehuda O Bøtker HE Maeng M Kjøller-Hansen L Identification of vulnerable plaques and patients by intracoronary near-infrared spectroscopy and ultrasound (PROSPECT II): a prospective natural history study Lancet 2021 Mar 13 397 10278 985 95 33714389 10.1016/S0140-6736(21)00249-X

[44] Oemrawsingh RM Cheng JM García-García HM van Geuns RJ de Boer SP Simsek C Near-infrared spectroscopy predicts cardiovascular outcome in patients with coronary artery disease J Am Coll Cardiol 2014 Dec 16 64 23 2510 8 25500237 10.1016/j.jacc.2014.07.998

[45] Ali ZA Karimi Galougahi K Mintz GS Maehara A Shlofmitz RA Mattesini A Intracoronary optical coherence tomography: state of the art and future directions Euro-Intervention 2021 Jun 11 17 2 e105 23 34110288 10.4244/EIJ-D-21-00089 PMC9725016

[46] Prati F Romagnoli E Gatto L La Manna A Burzotta F Ozaki Y Relationship between coronary plaque morphology of the left anterior descending artery and 12 months clinical outcome: the CLIMA study Eur Heart J 2020 Jan 14 41 3 383 91 Erratum in: Eur Heart J 2020 Jan 14;41(3):393 31504405 10.1093/eurheartj/ehz520

[47] Kedhi E Berta B Roleder T Hermanides RS Fabris E IJsselmuiden AJJ Thin-cap fibroatheroma predicts clinical events in diabetic patients with normal fractional flow reserve: the COMBINE OCT-FFR trial Eur Heart J 2021 Dec 1 42 45 4671 9 34345911 10.1093/eurheartj/ehab433

[48] Kolossváry M Szilveszter B Merkely B Maurovich-Horvat P Plaque imaging with CT-a comprehensive review on coronary CT angiography based risk assessment Cardiovasc Diagn Ther 2017 Oct 7 5 489 506 29255692 10.21037/cdt.2016.11.06 PMC5716945

[49] Conte E Annoni A Pontone G Mushtaq S Guglielmo M Baggiano A Evaluation of coronary plaque characteristics with coronary computed tomography angiography in patients with non-obstructive coronary artery disease: a long-term follow-up study Eur Heart J Cardiovasc Imaging 2017 Oct 1 18 10 1170 8 27679600 10.1093/ehjci/jew200

[50] Otsuka K Fukuda S Tanaka A Nakanishi K Taguchi H Yoshikawa J Napkin-ring sign on coronary CT angiography for the prediction of acute coronary syndrome JACC Cardiovasc Imaging 2013 Apr 6 4 448 57 23498679 10.1016/j.jcmg.2012.09.016

[51] Kröner ES van Velzen JE Boogers MJ Siebelink HM Schalij MJ Kroft LJ Positive remodeling on coronary computed tomography as a marker for plaque vulnerability on virtual histology intravascular ultrasound Am J Cardiol 2011 Jun 15 107 12 1725 9 21481832 10.1016/j.amjcard.2011.02.337

[52] Ferencik M Mayrhofer T Bittner DO Emami H Puchner SB Lu MT Use of high-risk coronary atherosclerotic plaque detection for risk stratification of patients with stable chest pain: a secondary analysis of the PROMISE randomized clinical trial JAMA Cardiol 2018 Feb 1 3 2 144 52 29322167 10.1001/jamacardio.2017.4973 PMC5838601

[53] Fairbairn TA Nieman K Akasaka T Nørgaard BL Berman DS Raff G Real-world clinical utility and impact on clinical decision-making of coronary computed tomography angiography-derived fractional flow reserve: lessons from the ADVANCE registry Eur Heart J 2018 Nov 1 39 41 3701 11 30165613 10.1093/eurheartj/ehy530 PMC6215963

[54] Leipsic J Yang TH Thompson A Koo BK Mancini GB Taylor Ce CT angiography (CTA) and diagnostic performance of noninvasive fractional flow reserve: results from the Determination of Fractional Flow Reserve by Anatomic CTA (DeFACTO) study AJR Am J Roentgenol 2014 May 202 5 989 94 24758651 10.2214/AJR.13.11441

[55] Lee JM Choi G Koo BK Hwang D Park J Zhang J Identification of high-risk plaques destined to cause acute coronary syndrome using coronary computed tomographic angiography and computational fluid dynamics JACC Cardiovasc Imaging 2019 Jun 12 6 1032 43 Erratum in: JACC Cardiovasc Imaging 2019 Nov 12:11 Pt 1:2288–2289 29550316 10.1016/j.jcmg.2018.01.023

[56] Gaur S Taylor CA Jensen JM Bøtker HE Christiansen EH Kaltoft AK FFR derived from coronary CT angiography in nonculprit lesions of patients with recent STEMI JACC Cardiovasc Imaging 2017 Apr 10 4 424 33 27743953 10.1016/j.jcmg.2016.05.019

[57] Ismail TF Strugnell W Coletti C Boẑić-Iven M Weingärtner S Hammernik K Cardiac MR: from theory to practice Front Cardiovasc Med 2022 Mar 3 9 826283 35310962 10.3389/fcvm.2022.826283 PMC8927633

[58] Patel AR Salerno M Kwong RY Singh A Heydari B Kramer CM Stress cardiac magnetic resonance myocardial perfusion imaging: JACC review topic of the week J Am Coll Cardiol 2021 Oct 19 78 16 1655 68 34649703 10.1016/j.jacc.2021.08.022 PMC8530410

[59] Everaars H van der Hoeven NW Janssens GN van Leeuwen MA van Loon RB Schumacher SP Cardiac magnetic resonance for evaluating nonculprit lesions after myocardial infarction: comparison with fractional flow reserve JACC Cardiovasc Imaging 2020 Mar 13 3 715 28 31542525 10.1016/j.jcmg.2019.07.019

